# Evaluating the Effects of Dithiothreitol and Fructose on Cell Viability and Function of Cryopreserved Primary Rat Hepatocytes and HepG2 Cell Line

**DOI:** 10.5812/hepatmon.7824

**Published:** 2013-01-22

**Authors:** Mahdokht H Aghdai, Akram Jamshidzadeh, Mahsa Nematizadeh, Mahtab Behzadiannia, Hossein Niknahad, Zahra Amirghofran, Elaheh Esfandiari, Negar Azarpira

**Affiliations:** 1 Transplant Research Center Transplant Research Center, Shiraz University of Medical Sciences, Shiraz, IR Iran; 2 Department of Pharmacology and Toxicology, Faculty of Pharmacy and Pharmaceutical Sciences Research Center, Shiraz University of Medical Sciences, Shiraz, IR Iran; 3 Department of Immunology, Shiraz University of Medical Sciences, Shiraz, IR Iran

**Keywords:** Hepatocytes, Cryopreservation, Fructose, Dithiothreitol

## Abstract

**Background:**

Hepatocytes are used as an in vitro model to evaluate drug metabolism. Human hepatocyte transplant has been considered as the temporary treatment of acute liver failure. Optimization freezing methods is very important to preserve both cell viability and function which are achieved by cryopreservation mostly always.

**Objectives:**

The present study aimed to investigate the cryoprotective effect of DTT and fructose on primary rat hepatocytes and HepG2 cells.

**Materials and Methods:**

Both fresh rat hepatocytes and HepG2 cell line were incubated with fructose (100 and 200 mM) and dithiothreitol (DTT) (25, 50, 100, 250, and 500 μM) at 37°C for 1 and 3 hours, respectively. The preincubated hepatocytes were cryopreserved for two weeks. Hepatocytes viability and function were determined post thawing and the results were compared with the control group.

**Results:**

The viability of both rat hepatocytes and HepG2 cells were significantly increased after one hour preincubation with fructose 200 mM. Preincubation with DTT (50 μM, 100 μM. 250 μM and 500 μM) improved the viability and function upon thawing in both cell types (P < 0.001). In rat hepatocytes, no significant change was observed in albumin, urea production, and LDH leakage after preincubation with fructose or DTT. In HepG2 cells, albumin and urea production were significantly increased after preincubation with DTT (500 μM, 1 hour). The GSH content was significantly increased in DTT (250 and 500 μM, 1 hour) groups in both rat hepatocyte and HepG2 cells.

**Conclusions:**

Incubation of hepatocytes with fructose and DTT prior to the cryopreservation can increase the cell viability and function after thawing.

## 1. Background

Optimization hepatocyte isolation methods and cryopreservation techniques are important to increase the viability of primary human hepatocytes for their clinical and preclinical applications. Human hepatocytes are usually used as an in vitro model in drug toxicity, metabolism and also in the cell therapy for hepatic failure. During human hepatocyte isolation, oxidative stress and cell death start after liver resection (1-3) and donor clinical condition, liver fat amount, and cold and warm ischemia times affect the quality of hepatocytes (4-6). During the isolation procedure, factors such as the time of procedure and the type of collagenase, can also determine the quality of cells (7-9). Several human hepatocyte cryopreservation protocols are currently in use (3-9). Hepatocyte is very susceptible to injury during freezing and thawing and its function usually deteriorates (10). In cryopreservation procedure, incubation with protective materials improves the hepatocyte function after thawing. Incubation in the culture media that contain glucose can improve the viability and energy status of the isolated hepatocytes (10, 11). This beneficial effect is due to the increase in cellular adenosine triphosphate (ATP) levels before cryopreservation which is depleted during cell isolation (10, 11). Dithiothreitol (DTT), with antioxidant properties, accelerates the decomposition of hydrogen peroxide in culture medium and prevents the cytotoxic effects of H_2_O_2_ (12, 13). Based upon a previous study, using DTT as a cryoprotectant improved the overall hepatocyte viability (14).

## 2. Objectives

The present study aimed to investigate the effects of preincubation of primary rat hepatocytes with DTT and fructose prior to cryopreservation. In parallel, HepG2 (Human hepatocellular carcinoma, cell line) was also examined. The cells viability and their function were subsequently evaluated after thawing.

## 3. Materials and Methods

### 3.1. Rat Hepatocyte Isolation

Sprague-Dawley male rats (200-250 g) were obtained from the Laboratory Animals Research Center of Shiraz University of Medical Sciences, Shiraz, Iran. Hepatocyte isolation was performed according to the collagenase perfusion procedure which was described by Reese et al. (15). Hepatocytes (1 × 106 cells/ml) were placed into Krebs-Henseleit buffer (pH: 7.4) containing 12.5 mM HEPES (Sigma-Aldrich, UK) and kept at 37 °C with 95% O^2^ and 5% CO^2^. Hepatocytes with a viability of more than 75%, which was measured with Trypan Blue (Sigma-Aldrich, UK), were used in the experiments.

### 3.2. HepG2 Cell Line Culture

The cell line was obtained from NCBI (Pasture Institute, Tehran, Iran) and grown in 75 cm^2^ cell culture flasks (NUNC, Germany) in RPMI medium supplemented with 10% FBS (Gibco, Germany), penicillin (50 U/ml), streptomycin (50 μg/ml) (Gibco, Germany), and L-glutamine (2 mM) (Gibco, Germany). The cells were maintained in a humidified atmosphere of 10% CO^2^ and 90% air at 37 °C. The culture medium was renewed every 2 to 4 days.

### 3.3. Hepatocyte Incubation, Cryopreservation and Thawing

Following the isolation, the hepatocytes were incubated with fructose (25, 50, 100 , 250, and 500 μM) (Sigma-Aldrich, UK) and DTT (100 and 200 mM) (Sigma-Aldrich, UK) in Williams’ culture medium E (WME) (Life Technologies, USA) at 37°C for 1 and 3 hours, respectively. The same experiments were applied to the HepG2 cell line. A control group (without preincubation) was also considered in each experiment for the comparison. Each experiment was repeated three times. About 2.5 × 106 cells/mL was resuspended in ice-cold freezing medium containing 10% DMSO (Sigma-Aldrich, UK), 50% FBS, and 40% culture medium (WME). The cells were transferred into cooled cryogenic vials and incubated on ice for 10 min. After that, the vials were placed into a controlled rate cooler (Mr. Frosty, UK) at -80°C and then transferred into liquid nitrogen (16). The cryopreserved cells were removed from liquid nitrogen (after two weeks), thawed in 37°C water-bath, washed with culture medium, and resuspended in fresh Williams’ medium E at a density of 106 cells/ml (14-16). The hepatocytes were placed into 96-well flat-bottomed collagen-coated plates (Thermo scientific, NUNC, USA) at 37°C and 5% CO^2^. All aforementioned tests were performed in triplicate.

### 3.4. Viability Assay

The cell viability was evaluated by Trypan Blue dye exclusion test.

### 3.5. Attachment Efficiency and Total Protein Measurement

The hepatocytes were cultured in gridded plates (2 × 2 mm) (Thermo scientific, NUNC, USA) containing Williams’ culture medium, 10% FBS with penicillin/streptomycin and L-glutamine. After 24 hours, the percentage of attached hepatocytes was calculated after counting with inverted light microscope (Olympus Ltd., Japan). Besides, the total protein concentration was measured according to the Bradford protein assay.

### 3.6. Function Assays

The functional assays were evaluated with 3×104 viable cells/well in collagen-coated plates. After 24 hours, the concentrations of LDH, albumin, and urea were determined. The concentration of LDH was determined in supernatant using a commercial Quantification Kit protocol (Pars Azemun, Tehran, Iran) (17-19). In addition, albumin and urea concentrations were determined using a commercial Quantification Kit (Kimia Pajuhan, Tehran, Iran).

### 3.7. Determination of Reduced Glutathione (GSH)

Reduced glutathione was measured using the glutathione reductase 5, 5'-dithiobis-2-nitrobenzoic acid (DTNB) recycling protocol (20, 21).

### 3.8. Statistical Analysis

All the results are presented as mean ± SD. Statistical analysis of the data was performed by comparing the means through one-way ANOVA and Posttest (Dunnett). All statistical analyses were performed using the SPSS statistical software (v. 15.0, Inc., Chicago. IL, USA) and P values ≤ 0.05 were considered as statistically significant. The figures were drawn by using Graph Pad Prism version 5.

## 4. Results

### 4.1. Cell Viability

**Figure 1:**
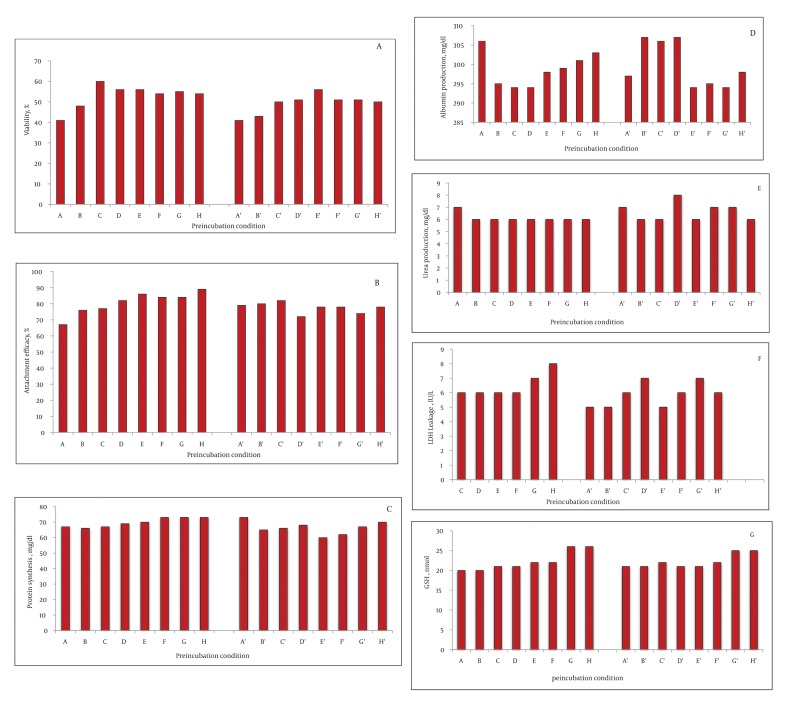
The Effect of Rat Hepatocytes Preincubation with Fructose and DTT Viability, Attachment Efficacy, Functional Assessment and Reduced Glutathione Were Measured. The hepatocytes were incubated with fructose (100 μM (B,B'), 200 μM (C,C') and DTT (25 μM (D,D'), 50 μM (E,E'), 100 μM (F,F'), 250 μM (G, G'), 500 μM (H,H'). The control group was (A, A'). The preincubation time was one hour (A-H) and three hours (A'-H'). Statistical significance was considered if P < 0.01. Panels A to G show A) the viability after thawing; B) attachment efficacy (%); C) protein synthesis (mg/dl); D) Albumin production (mg/dl); E) Urea production (mg/dl); F) LDH leakage (IU/L) and G) GSH content (nmol).

**Figure 2:**
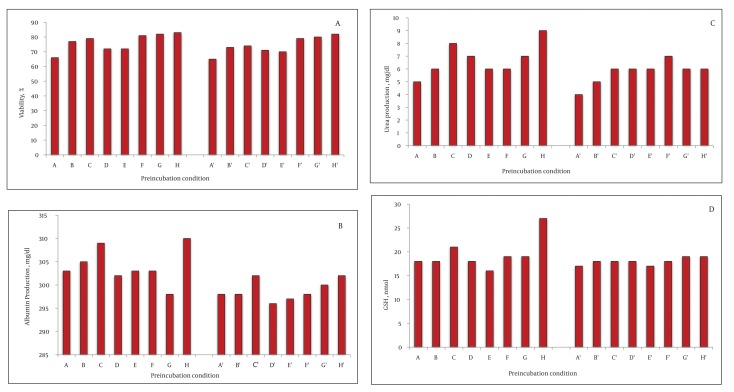
The Effect of HepG2 Cell Line Preincubation with Fructose and DTT. Viability, Functional Assessment and Reduced Glutathione Were Measured. The hepatocytes were incubated with fructose (100 μM (B, B'), 200 μM (C, C') and DTT (25 μM (D, D’), 50 μM (E, E’), 100 μM (F, F’), 250 μM (G, G'), 500 μM (H,H'). The control group was (A, A'). The preincubation time was one hour (A-H) and three hours (A'-H'). Statistical significance was considered if *P < 0.01. Panels A to D show [A] the viability after thawing; [B] Albumin production (mg/dl); [C] Urea production (mg/dl) and [D] GSH content (nmol).

The viability of fresh isolated rat hepatocytes was 86% to 98%. On the other hand, the viability of the control cryopreserved hepatocytes after thawing was 44 ± 4% after two weeks storage in liquid nitrogen. Preincubation with fructose (200 mM) for one and three hours increased the viability after thawing significantly compared to the control group (200 mM = 55 ± 2 %, P = 0.003, 51 ± 1 %, P = 0.004). DTT preincubation showed a significant effect, as well (DTT 25 μM = 49 ± 1%, P = 0.003; DTT 50 μM = 55 ± 5%, P = 0.004; DTT 100 μM = 49 ± 1%, P = 0.004; DTT 250 μM = 50 ± 4%, P = 0.003; DTT 500 μM = 53 ± 48%, P = 0.003). The three hour preincubation of the rat hepatocytes with these substances also increased the viability after thawing significantly (P < 0.001), (Figure 1A). Nevertheless, comparison of the viability after thawing showed that the values at three hours were lower than one hour incubation. With respect to HepG2 cells, one hour incubation with fructose (200 mM) increased the cell viability after thawing (77 ± 2, P = 0.004). The viability after thawing of the cells was also increased after preincubation with DTT (100, 250, and 500 μM) for both one and three hours (P < 0.001) (Figure 2A). Although the viability after thawing was increased in both one and three hours separately, the values at three hours were lower than one hour incubation.

### 4.2. Attachment Efficacy and Protein Synthesis

In the rat hepatocyte, the attachment efficacy and protein synthesis after thawing were significantly increased after one hour preincubation with DTT 25, 50, 100, 250, and 500 μM (P < 0.001). However, no significant concentration-dependent effect was detected after incubation with fructose (100 and 200 mM) (1 or 3 hours) and DTT (3 hours) in the rat hepatocyte (Figure 1B and C).

### 4.3. Function Assay After Thawing

No significant change was observed in albumin, urea production, and LDH leakage after incubation of the rat hepatocytes with fructose or DTT (Figures 1D, E and F). In HepG2 cells, albumin secretion was significantly increased after preincubation with fructose (200 mM, 1 hour, P = 0.004) and DTT (500 μM, 1 hour, P = 0.04) (Figure 2B). The urea synthesis was also increased after preincubation with fructose (200 mM, 1 hour, P = 0.001) and DTT (500 μM, 1 hour, P = 0.01) (Figure 2C).

### 4.4. Glutathione Content After Thawing

The GSH content was significantly increased with DTT preincubation (250 and 500 μM, 1 hour) in both rat hepatocyte (Figure 1G) and HepG2 cells (Figure 1E) (P < 0.001). Of course, the values at three hours were lower than one hour incubation (Figure 2D). Furthermore, after thawing, the values of attachment efficacy, protein synthesis, functional assay, and glutathione content at three hours were lower than one hour incubation.

## 5. Discussion

Cryopreservation is increasingly used for long-term storage of viable cells and tissues. However, both freezing and thawing processes result in severe damages and tissue injury (22, 23). Different mechanisms, such as oxidative stress, ice crystal formation with osmotic injury, activation of caspase-3 with apoptosis and disturbed ion homeostasis due to Na + /K+-ATPase pump inhibition, are responsible for cell damage during freeze-thaw processes (24, 25). Cryoprotective substances, such as dimethyl sulfoxide (DMSO), glycerol, ethylene glycol and hydroxyethyl starch, protect cells from dehydration and prevent lethal intracellular ice formation which occurs during freezing (23). However, these agents are toxic for cells and are needed to be rapidly eliminated after freezing (26). DMSO also causes an osmotic stress which affects cells metabolic activity (23, 27). Therefore, cryopreserved cells do not have the metabolic state of fresh cells. Functional activity of major enzymes which are involved in hepatocyte metabolism maintains after cryopreservation except in case of rapid activity loss of cytochrome P450 (P450) (10, 28). To eliminate this problem, sugars and caspase inhibitor have been used to increase the stability of the hepatocyte (10, 29, 30). Grondin et al. revealed that using crude wheat protein extract as a cryoprotective substance improves the hepatocyte specific functions and allows hepatocytes to be maintained in culture for 4 days (31). Its effect was due to the presence of a mixture of materials, such as sugars and antioxidants, in the plant extract (31). Hepatocyte viability was evaluated immediately after isolation by using the standard Trypan Blue exclusion technique. The cells which excluded Trypan Blue were considered as “viable” and cells staining blue were “dead.” In this assay, false negative result was occurred. In fact, when cells have intact cell membranes, but have initiated apoptotic pathways, they might not stain with Trypan Blue but they are ultimately dead (30, 31). Despite its common use, it is not able to detect the cells undergoing apoptosis and, on the other hand, in vitro cell viability assay cannot predict the cell function after transplantation (32). There are other alternative tests which can be used to evaluate the viability and activity of the isolated hepatocytes, such as MTT assay, measurement of cytochrome P450 activity, protein synthesis assay using [14C]-leucine incorporation, DNA synthesis assay using incorporation of [3H]-thymidine, and measuring hepatic marker proteins such as albumin, transferrin, and apolipoproteins (32). These tests are believed to be more sensitive than Trypan Blue and reflect cells activity and function much better (32). However, these methods are usually suitable for attached cells. If cell suspension is used, the major problem is to determine the dead cells. Ideally, viable cells must be separated from the dead ones by the Percoll gradient centrifugation and then tested with the aforementioned assays (28). In fact, the Percoll gradient resulted in lower yield along with higher viable cells, which was suitable for any cell experiment (31, 32). These methods are time consuming and decline the cell viability (32). In our study, the viability after thawing of the control cryopreserved hepatocytes was 44%. This finding is in line with the study performed by Terry et al. reporting 45% viability after thawing of the control group (33). In the current study, Williams’ culture medium E was used in freezing medium, while in the literature, University of Wisconsin solution (UW solution) has been recommended (13, 32, 33). The study results showed that using fructose improves the viability after thawing of the cells (both primary cells and cell line). After preincubation with DTT (250 and 500 μM), the cell viability, attachment efficacy and function were significantly increased. Terry et al. have shown that incubation of hepatocytes with glucose, fructose, or α-lipoic acid prior to freezing improves the post thaw viability and function of the hepatocytes (33). Fructose can protect the hepatocytes against apoptosis by forming nicotinamide adenine dinucleotide phosphate (NADPH) to regenerate the reduced glutathione (GSH). This event reduces the generation of reactive oxygen species and affects the survival (34). Using fructose during hepatocyte isolation improves the recovery of energy by cells after ischemia reperfusion injury and cell isolation stress (33). GSH as an important component of cellular antioxidant defense mechanism, plays a crucial role in protecting cells against oxidative stress, and is essential for cell functions and survival. DTT directly reduces the thiol groups and protects the cells against oxidative damage (35, 36). Intracellular GSH content was significantly depleted before cell death and these cells were more sensitive to damage when exposed to toxic chemicals (35, 36). Stevenson et al. found that during cryopreservation, the intracellular reduced glutathione was significantly decreased and adding ascorbic acid and α-tocopherol improved the content of reduced glutathione in postcryopreserved hepatocytes during freezing (37, 38). The findings of the current study indicated that prolonged incubation with DTT was toxic for the cells; therefore, lower concentration with short preincubation is recommended. Moreover, the freeze-thaw process was associated with caspase activation and apoptosis had an important role in the cryoinjury of the cells. Stroh et al. found that using caspase inhibitors, such as zVAD-fmk, improves the recovery and survival of the cryopreserved cell lines and hematopoietic progenitor cells. They revealed that adding zVAD-fmk to both the freezing solution and the culture medium was protective during the thaw process (39). Gauthaman et al. showed that using Rho-associated kinase (ROCK) inhibitor Y-27632 inhibited the apoptosis and increased the proliferation of the frozen-thawed human umbilical cord Wharton's jelly stem cells (40). In addition, Y-27632 increased the survival of human embryonic stem cells after thawing significantly (41). In this study, we used fresh rat hepatocytes and human HepG2 cell line to compare the primary cell and the cell line. However, it would have been more appropriate to use a human immortalized normal hepatocyte cell line, such as HepaRG cells, which retains many characteristics of the primary human hepatocytes. Using caspase inhibitor or ROCK inhibitor may improve the survival of hepatocytes after thawing. Of course, performing further experiments with other cryoprotective agents for cryopreservation of the primary human hepatocyte is important for the success of cell and tissue transplantation as well as tissue engineering. Cryopreservation is important for hepatocyte storing, but adversely affects the function and cell viability after thawing. Preincubation with fructose and DTT prior to cryopreservation increase both cell viability and hepatocyte function upon thawing. Of course, further studies are recommended to evaluate the effect of these substances on viability and function of the human hepatocytes.
